# ‘As Important as Medication’. A Qualitative Investigation of the Beliefs, Barriers and Facilitators of Physical Activity for Women With Metastatic Breast Cancer

**DOI:** 10.1002/pon.70193

**Published:** 2025-05-28

**Authors:** Zainab Faatimah Haider, Rebecca J. Beeken, Anam Ayaz‐Shah, Kelly E. Lloyd, Samuel George Smith

**Affiliations:** ^1^ Leeds Institute of Health Sciences University of Leeds Leeds UK

**Keywords:** cancer, exercise, metastatic breast cancer, oncology, physical activity, quality of life, reflexive thematic analysis, Theoretical Domains Framework

## Abstract

**Background:**

Women with metastatic breast cancer (MBC) experience a range of physical and psychological challenges. Physical activity is safe and supports quality of life in individuals with MBC. However, most women with MBC are not meeting physical activity recommendations.

**Aims:**

To inform the development of an accessible and effective physical activity intervention, we investigated the beliefs, barriers and facilitators of physical activity of women with MBC.

**Methods:**

We conducted semi‐structured telephone interviews with UK women living with MBC. Participants were asked open‐ended questions about physical activity levels, beliefs, barriers, facilitators, support and preferences. Reflexive thematic analysis was used to analyse the transcripts and themes were mapped onto the Theoretical Domains Framework.

**Results:**

23 women with MBC (mean age = 53.7 years; White British = 69.7%) were interviewed between May 2022 and October 2024. Average time since diagnosis was 6 years, 1 month. Most had bone metastases (68.8%). Our analysis generated five themes. Participants generally believed physical activity was important (1) but were limited by physical symptoms (2) and psychological barriers (3). Most women noted a significant lack of physical activity support from healthcare providers (4). They discussed how physical activity could be more accessible for their diverse needs (5), including increasing provision of tailored support.

**Conclusions:**

Improvements in physical activity support for women with MBC are needed. Our findings provide several implications for future interventions. This includes implementing physical activity recommendations, improving knowledge about risks and benefits, and incorporating psycho‐social and tailored support which consider the unique needs of the MBC population.

## 1 Background

In the UK, an estimated 57,000 individuals were living with metastatic breast cancer (MBC) between 2020 and 2021 [1]. Treatment advances have led to greater life expectancy and more women living with MBC [2, 3]. Many undergo treatment long‐term and consequently experience symptoms like pain, fatigue and nausea, as well as an increased risk of bone metastases [4, 5]. Managing the uncertainty of how long treatments will be effective can result in depression and anxiety [6]. Supporting quality of life in women with MBC is increasingly important.

Well‐established literature highlights the benefits of physical activity for women with early‐stage breast cancer, including improving symptoms and wellbeing [7, 8]. There is less evidence in women with MBC. Recent systematic reviews conclude physical activity is safe and can be feasible for individuals with metastatic cancer, including MBC [9, 10, 11]. Physical activity may also support symptom management [12, 13]. In 2024, the largest trial to date (*n* = 357) evaluated the effectiveness of a supervised exercise intervention for women with MBC [14]. The intervention group showed significant and clinically relevant improvements in fatigue and quality of life outcomes like pain, physical functioning and dyspnea. The intervention was also cost‐effective [15].

While physical activity is likely to be beneficial for women with MBC, many are not active [5, 16]. No physical activity programme currently exists within the UK National Health Service (NHS) specifically for women with MBC, whose physical and psychological needs differ from early‐stage breast cancer [17, 18]. As the number of women living with MBC increases, so does the need for support.

To inform the development of accessible and effective physical activity interventions, there is a need to understand the beliefs, barriers and facilitators of physical activity in women with MBC [19, 20]. Two European focus group studies (non‐UK), found individuals with MBC held positive attitudes towards exercise, but physical barriers limited their participation in programmes not tailored to their needs [19, 21]. However, these studies explored attitudes towards supervised exercise only. Non‐supervised and less structured physical activity may be more accessible for populations who struggle to be active [22, 23]. To our knowledge, only one study has explored perceptions and barriers to non‐supervised physical activity in the MBC population. In this Australian study, most participants agreed physical activity was important but wanted a more nuanced and individualised approach to promoting physical activity in their daily lives [24, 25]. No UK study has explored beliefs, barriers and facilitators of physical activity for women with MBC.

A further limitation to the existing literature is the lack of theoretical frameworks applied during intervention design. Theory helps identify influences on behaviour that are amenable to change, and thus inform later intervention development [26]. The Theoretical Domains Framework (TDF) integrates multiple behaviour change theories, and is comprised of 14 domains that could impact behaviour [27]. The breadth of the TDF makes it suitable for a patient group with diverse and complicated physical, psychological and environmental contexts [28]. A flexible approach to using the TDF has also been promoted for exploratory qualitative research [28]. For example, after an initial, inductive analysis of the data to capture rich data, the TDF can be used as a deductive framework to interpret the findings.

We conducted a qualitative interview study to explore the beliefs, barriers and facilitators of physical activity among UK women with MBC. We used reflexive thematic analysis for an in‐depth, inductive exploration of patient experiences, before mapping themes to key domains of the TDF. This use of theory complemented the richness of the qualitative data while also allowing us to identify influences on physical activity to be targeted in future interventions.

## 2 Methods

### 2.1 Participant Recruitment

Eligibility criteria included (1) self‐reported diagnosis of MBC; (2) over 18; (3) lived in the UK; (4) provided informed consent; (5) could hold a conversation in English, specifying that English did not need to be their first language. All participants were emailed an information sheet and were asked to sign a consent form. The information sheet was assessed against the Flesch‐Kincaid calculator to maximise readability. Participants were compensated with a £25 voucher.

Participants were recruited using two routes. First, we recruited people who took part in the Advancing Survival After Cancer Outcomes Trial (ASCOT), which aimed to improve health behaviours among individuals living with and beyond cancer [29]. We contacted all those who indicated they (1) had breast cancer, (2) at follow‐up, the cancer had ‘spread to other parts of the body’, and (3) consented to take part in further research. Demographic data was collected as part of the wider trial.

Second, we recruited women with MBC via social media (x, formally Twitter) local support groups and cancer charities, prioritising participants from ethnic minority or non‐University educated backgrounds. We asked participants to share the opportunity with their networks. All participants recruited via this route completed a demographic and clinical questionnaire. We used an established approach for assessing data saturation in theory‐based interview studies [30]. This allowed us to balance the need for quantity and quality of data, whilst minimising the risk of resource waste and participant burden. We specified an initial analysis sample of 20 a priori and determined data saturation was achieved when three consecutive interviews generated no new themes.

### 2.2 Data Collection

ZFH conducted all interviews over the telephone. ZFH is a female postgraduate researcher trained in qualitative interviewing and analysis, working with underrepresented groups and sensitive communication. She has experience running workshops and focus groups with MBC patients. All authors are part of a behavioural oncology research group which has an interest in evaluating physical activity interventions for cancer survivors. They generally have positive views about the benefits of physical activity in the breast cancer context.

The semi‐structured interviews included open‐ended questions about physical activity (1) levels; (2) beliefs and attitudes; (3) barriers and facilitators; (4) support; (5) and preferences. These were chosen to inform intervention development in the future. ZFH kept a field diary while interviewing, but this was used reflexively during analysis and was not part of formal data collection.

### 2.3 Data Analysis

Interviews were audio recorded. They were transcribed verbatim by an external company contracted by the University and were adherent to UK General Data Protection Regulation. All transcripts were pseudonymised before undergoing analysis, with each participant given randomly selected initials as identifiers. Reflexive thematic analysis was used to analyse the transcripts, using an inductive approach [31]. All interviews were analysed by ZFH and five interviews were coded by another researcher, AAS, to enhance interpretative depth of the data by discussing different perspectives, rather than to confirm findings [32].

First, we familiarised ourselves with the data by actively reviewing the transcripts and cross‐referencing with the field diary. Second, all transcripts were coded inductively. Codes were grouped into the broad topics included in the interview schedule (physical activity levels, beliefs, barriers, facilitators, support and preferences). Third, themes were actively generated from these code groups based on shared patterns of meaning and refined in several iterations [31]. Finally, we mapped each sub‐theme to the most relevant TDF domain, which provided a deductive and interpretive framework to help identify implications for future interventions.

ZFH and AAS met three times to discuss the coding approach, initial codes and the development and refinement of themes. ZFH mapped the themes onto the TDF. SS, RB and AAS reviewed the final themes and TDF mappings.

## 3 Results

Twenty‐three eligible participants returned the consent form and took part in an interview between 19^th^ May 2022 and 28^th^ October 2024. Of these participants, seven were recruited from the ASCOT trial, and 16 from the second wave of recruitment. Demographic and clinical characteristics are presented in Table 1.

**TABLE 1:** Participant demographic and clinical characteristics.

	Mean (SD)	*N*	%
Age (years)^a^	53.7 (9.1)		
40–45		4	18.2
46–50		6	27.3
51–55		2	9.1
56–60		2	9.1
61–65		7	31.8
66–70		1	4.5
Ethnicity			
White british		16	69.6
White (other)		2	8.7
Mixed ethnicities		1	4.3
Indian		1	4.3
Black caribbean		3	13.0
Employment status			
Full‐time work		5	21.7
Part‐time work		4	17.4
Unemployed		2	8.7
Too sick to work		2	8.7
Retired		9	39.1
Other		1	4.3
Highest level of education			
GCSE		2	8.7
National Vocational Qualification (NVQ)		2	8.7
A level		3	13.0
Degree/Above		16	69.6
MBC Sub‐type^b^			
HER‐2 Positive		7	43.8
HER‐2 Negative		1	6.3
HER‐2 Negative/ER+		3	18.8
Triple Negative		1	6.3
Luminal		3	18.8
Other		1	6.3
Line of therapy^b^			
First		8	50.0
Second		4	25.0
Third and above		4	25.0
Years since diagnosis^b^	6 years 1 month		
Less than 1 year		2	12.5
1–3 years		3	18.8
3–6 years		7	43.8
6–9 years		1	6.3
9–12 years		1	6.3
12+ years		2	12.5
Cancer spread^b^			
Bone		11	68.8
Lymph nodes		6	37.5
Liver		9	56.3
Lung		3	18.8
Brain		3	18.8
Treatments received			
Chemotherapy		17	73.9
Radiotherapy		12	52.2
Surgery		16	69.6
Hormone therapy		13	56.5
Biological agents		9	39.1
CDK4/6 inhibitors		8	50.0
Immunotherapy		2	12.5
Holistic therapy		3	18.8

*Note:* Demographic and clinical information for the first sample was only collected as part of the wider trial. This meant the clinical information collected in the second sample via a questionnaire, were not available for the first sample.

Abbreviation: SD, standard deviation.

^a^ Data was missing from one participant.

^b^ Data collected from second sample only.

Our analysis generated five main themes: (1) Positive beliefs and knowledge about physical activity; (2) Physical ability limits physical activity; (3) Important psychological aspects of physical activity; (4) Levels of support from HCPs and charities and (5) Making physical activity more accessible. Table 2 provides an overview of these themes and their sub‐themes, mapped onto TDF domains.

**TABLE 2:** Overview of themes and sub‐themes, mapped onto the Theoretical Domains Framework (TDF).

Main theme	Sub‐themes	Main TDF domain(s)
3.1 Positive Beliefs and Knowledge about Physical Activity	Recognising the importance of physical activity	Intentions
	Experiencing the benefits of physical activity	Beliefs about consequences Reinforcement
	Having knowledge about physical activity	Knowledge Beliefs about consequences
	Having a balanced view of physical activity in life	Optimism
3.2 Physical Ability limits Physical Activity	Physical symptoms limit physical activity	Environmental context and resources Skills
	Diversity of experiences affected physical activity levels	Environmental context and resources
	Fearing an injury limits physical activity	Emotion Beliefs about consequences Beliefs about capabilities
	Fearing an infection limits physical activity	Emotion Beliefs about consequences
3.3 Important Psychological Aspects of Physical Activity	Poor mental health as a barrier	Emotion
	Lack of confidence about capabilities and body image limits physical activity	Beliefs about capabilities Emotion Social influence
	Having low motivation to exercise	Intentions Goals Optimism
	Physical activity helps with wellbeing	Emotion Beliefs about consequences Reinforcement
	Importance of psychological support for physical activity	Social influence Environmental context and resources
	Importance of social support for physical activity	Social influence
3.4 Levels of support from HCPs and charities	Lack of physical activity support from HCPs	Environmental context and resources Knowledge
	Supportive HCPs facilitates physical activity	Environmental context and resources
	Charities play a key role	Environmental context and resources
3.5 Making Physical Activity more Accessible	Bringing physical activity into the day makes it easier to maintain	Intentions Behavioural regulation
	Making physical activity fun helps with motivation	Social influence
	Setting reasonable goals helps maintain physical activity	Goals
	Need for tailored support which is specific for MBC	Environmental context and resources
	Need for tailored support which accounts for individual differences	Environmental context and resources
	Physical activity should be considered within a holistic approach to health	Environmental context and resources

### 3.1 Positive Beliefs and Knowledge About Physical Activity

Most participants believed physical activity was important and they tried to remain active.

Physical activity needs to become as important as whatever medication you give to a cancer patient.

RT

One key reason was experiencing the benefits of being active. Participants reflected on how physical activity helped with their symptoms and treatment side‐effects, for example, with energy, strength, sleep, digestion, and muscle pain.

When I was doing my jogging group, I used to be on a real high afterwards…I’d have so much energy, even though I’d just ran.

KF

Participants also discussed their knowledge and beliefs about physical activity in the context of cancer, for example preventing recurrence and promoting longevity.

I stick to this training programme quite a lot…probably because I think that this keeps my cancer at bay.

ZL

However, many reflected on the balance of physical activity in their lives. Participants noted it was not the most important thing, nor should they feel guilty for struggling.

Our life is so up and down and it’s so unpredictable that it’s hard to commit to something when there are already lots of commitments…it’s not the most important commitment. (They should) not feel guilty if they can’t do it or are too tired or have got something more important on.

EB

### 3.2 Physical Ability Limits Physical Ability

Participants were diverse in their physical capability levels. Some reported no limits, but most discussed struggling to some degree with being active. Participants consistently reported physical symptoms made activity more challenging. Fatigue and pain were most commonly reported, but breathlessness, neuropathy, muscle stiffness, lymphoedema, hand and foot syndrome and nausea were all discussed.

I would get a lot of joint pain so sometimes I would struggle to walk, even getting out of bed would be hard…I did suffer from very sore knees, very sore ankles and sore elbows and so just doing physical things was quite painful at times.

EB

There was diversity in participant treatment experiences depending on what they received, or disease progression. Physical symptoms appeared worse during chemotherapy. Moving onto treatments with fewer side effects was a facilitator of physical activity. Those living with MBC for longer also noticed a gradual decrease in their physical abilities.

(My cancer) has progressed steadily… I am constantly on treatment. So, my energy levels and my pain levels by now have markedly deteriorated and stop me being active.

RW

Physical symptoms contributed to health‐related fears. Many were afraid they would injure themselves because they were recovering from surgery or had bone metastases. Additionally, because their cancer treatments compromised their immunity, some felt anxious about attending indoor exercise classes, going to gyms or swimming, fearing an infection.

I just withdrew into myself…I didn't feel that I could (exercise) because I was scared of hurting myself… I'd like to pick up more activity, but I'm scared.

RG

### 3.3 Important Psychological Aspects of Physical Activity

Many participants discussed the devastating effects of their diagnosis on their mental health, which acted as a barrier.

There’s a limit to what I could have done even if I’d been physically well enough to do it…Mentally you’re down because of the whole shocking situation… it was pretty tough to deal with, so physical activity wasn’t in the remit.

TE

Their reduced physical ability made them feel upset, disheartened, embarrassed and angry. Many found themselves comparing their ability to pre‐diagnosis or to other healthy women, reducing their desire to exercise in public. They talked about a lack of confidence and body image, especially if they had undergone a mastectomy. Not seeing representation of women who looked like them made them feel gyms and exercise classes were not for them.

It was a bit disheartening. I ended up stopping that yoga class, because I felt mentally it was making me feel bad, that I couldn’t do the things that I did before.

DG

I would feel obligated to wear my prosthetics and it’s uncomfortable to exercise wearing false boobs…When you’re in a gym, you’re wearing tight‐fitting clothing. There’s no hiding.

RW

As a result, low motivation to engage in activity was highlighted. Physical activity also felt harder, so some did not enjoy high‐intensity exercise. Not being able to meet their goals acted as a barrier.

I’m struggling with trying to motivate myself to do things because everything seems so much of an effort really when you’re suffering with fatigue and feeling quite down.

GH

However, nearly all women agreed being active supported their wellbeing, for example helping with stress, difficult feelings about their diagnosis and to feel ‘normal’. The combination of physical activity and being outdoors in nature was highlighted by a significant proportion of participants. The strong, positive effects physical activity had on wellbeing acted as a facilitator, as they knew it would make them feel better.

I would feel proud of myself after and it would lift my spirits a little bit…That’s why every day I'm so keen to just try and do something, because I would feel like I would be losing everything if I lost that as well.

EW

Consequently, psychological support also acted as a facilitator with supportive environments helping them to overcome psychological barriers. Social support was consistently highlighted as a way to make physical activity feel less like a ‘chore’ and address isolation, especially with others who understood what they were experiencing.

Social support and physical activity combined, I think could be a really good call… so people feel supported together, but at the same time, seeing the physical activity worthwhile.

CS

### 3.4 Levels of Support From HCPs and Charities

Nearly all participants noted they did not receive enough support with physical activity, and their healthcare provider (HCP) did not recommend it. Some reported their HCP actively discouraged physical activity because of risk of injury. Local and accessible opportunities to engage in physical activity appeared difficult to find which made them unsure about what they could do. Participants believed that support should be provided by NHS.

I was scared of doing too much physical activity because I just didn't get any good advice from any of my care team…when I asked what I could do, physical activity‐wise, it was just, “Oh, don't know, probably best not to do anything”.

PD

Despite the majority feeling they had not received enough support, some participants reflected on good examples of care, which facilitated physical activity.

The nurse that I saw just said, “It is really important that you do some physical activity, it doesn’t matter what it is…so that your mind is not just going over and over what is wrong with you,” and I found that very helpful advice.

RT

Additionally, the role of charities were positively highlighted in encouraging physical activity which appeared to fill a much‐needed hole in service provision.

Penny Brohn (charity) feel strongly about it when the hospitals don’t. They medicate you and they keep, monitor you, but they don’t offer you or suggest things which would make your life easier and better. You rely on charities to help you with that sort of thing.

GH

### 3.5 Making PA More Accessible

Participants discussed ways that physical activity could be made easier and accessible. First, by making it a part of their day, such as going on the school run, gardening, housework, walking to the hospital, or walking the dog. These activities were easier to maintain as they became routine.

(It’s) suggesting other ways of getting physical exercise. I’ve joined a local gardening group that does community gardening and makes our local area look nice, so I’m actually getting physical exercise through doing.

EB

Participants who struggled with motivating themselves suggested making physical activity fun, for example by combining it with socialising, music or a visit to a coffee shop.

Some reflected on setting reasonable goals and tangible plans. Making plans to be active, helped participants to remain active. Several participants owned a smart watch or a phone app which was used to set goals. Setting their own goals which considered their complex needs was helpful, but when goals were unattainable, they had the opposite effect.

A small goal that you can perhaps reach and then move on to the next goal, it certainly does help you.

EW

Nearly all participants discussed the need for tailored support, reflecting on the unique and diverse experiences of women with MBC. This was two‐fold. First, patients reported a lack of specific service provision for women with MBC specifically. General services were not sufficient, and even cancer‐specific programmes often ignored the unique needs of secondary cancer.

Most personal trainers, Pilates teachers, yoga teachers do not really understand the limitations of people with any kind of metastatic cancer because that often goes to the bones…(they) do not seem to understand the consequences of that.

RW

Second, participants expressed need for support which accounted for the differences within the MBC population, reflecting on the diversity of capabilities, and how needs change over time. Staged programmes which had different options depending on the ability of the individual were mentioned by several participants.

(Charities) have got somebody trained to tutor people with different levels of ability due to cancer and they’re very good at offering alternative types…They usually demonstrate something at a lower level for those who haven’t got the same amount of movement capabilities.

GH

Finally, conversations about tailored interventions brought up wider discussions about taking a holistic approach to their health. Participants argued that physical activity should be offered as a core part of treatment, along with other lifestyle and psychological support.

(We need to) treat people in holistic ways rather than just a lump of meat with a pain…Yes, we have to have medications and drugs but incorporate a little sort of plan for the individual, because that’s what we need.

OR

## 4 Discussion

In this UK interview study, women with MBC acknowledged the wide‐ranging benefits of physical activity, but experienced complex physical, psychological and healthcare barriers which limited their ability to engage in it. We showed rapid improvements in physical activity provision for this population are needed in the UK, with most participants not receiving appropriate advice from their HCPs. Our findings also align with those from mainland Europe and Australia [19, 21, 24], suggesting our findings may also generalise further than a national context. Overall, they emphasise that women with MBC require physical activity support which is tailored to their diverse and unique needs. Physical activity programmes which actively consider their physical limitations and incorporate psychological and social support are likely to be more accessible.

### 4.1 Clinical Implications

By mapping our sub‐themes to the domains of the TDF, we identified potential influences on physical activity to be targeted in future interventions for the MBC population.

Nine sub‐themes aligned with Environmental Context and Resources, which incorporated factors relating to the patient (e.g., ability to be active, physical symptoms) and their external support (e.g., lack of healthcare support, tailored physical activity). Our findings suggest increasing the availability and type of support offered to MBC patients would be an effective facilitator of physical activity. The implications are two‐fold. First, we suggest HCPs should lead more positive discussions about physical activity within consultations. Many participants experienced hesitation from HCPs to recommend physical activity, leading to uncertainty about how to perform activity safely within their physical limitations. However, HCPs have expressed a need for further clinical guidance around physical activity for people with metastatic cancer to feel more confident in prescribing it [33]. A recent positive step has been the publication of the first guidelines for exercising with bone metastases in 2022 by the International Bone Metastases Exercise Working Group [IBMEWG] [34]. The IBMEWG encompasses an international group of researchers and clinicians in exercise oncology, aiming to improve the clinical guidance around exercise for people with metastatic cancer. Further efforts are required to ensure these best practice recommendations are implemented confidently by all HCPs who work with MBC patients, to ensure they receive appropriate advice.

Second, our findings suggest tailored physical activity programmes which consider the needs and preferences of this patient group are likely to be useful. This aligns with other qualitative and quantitative research undertaken within this population [14, 19, 35]. One study explored MBC patient preferences for types of physical activity programmes, finding distinct classes of preferences based on clinical and demographic differences [35]. For example, preference for moderate, supervised exercise was most common among participants with bone metastases, whereas active walking was preferred by those without bone metastases. Tailored interventions may be difficult to translate into routine care. However, some participants in our study discussed how including different options for different abilities within a standard programme, and ensuring any supervision is sensitive of specific needs, could be sufficient.

Five sub‐themes aligned with Beliefs about Consequences. Knowing and experiencing physical and psychological benefits facilitated being physically active but negative beliefs about the consequences of physical activity—fear of injury and infection—acted as barriers. Improving knowledge about the benefits and risks of physical activity could help empower patients with MBC, help correct erroneous beliefs and facilitate physical activity. To improve confidence and help reinforce physical activity behaviour among non‐active patients, HCPs could also encourage them to engage in smaller bouts of physical activity to help them experience the benefits for themselves. Additionally, greater provision of supervised exercise by trained professionals, which was expressed as a preference by several participants, could help improve confidence to perform activity safely.

Four sub‐themes aligned with Emotion, with fear, depression and low self‐confidence acting as barriers to physical activity. These emotions are commonly experienced by individuals with MBC, and have been shown to negatively impact levels of physical activity [36, 37]. Our findings suggest incorporating psychological support into a physical activity programme could help address these psychological barriers. For example, supporting poor self‐esteem and confidence to be active, as well as providing wider therapeutic strategies for depression and low motivation. Incorporating psychological support into physical activity programmes for breast cancer survivors have already been shown to be effective at improving exercise adherence [38]. Similarly, given physical activity has a positive effect on wellbeing, HCPs could recommend physical activity as part of a holistic support programme, which is likely to improve quality of life in general.

Four sub‐themes reflected aspects of the social environment which could impact physical activity. The importance of having social support was emphasised, which supported participants' motivation, enjoyment and discipline regarding physical activity. A systematic review reported the importance of social support in enabling and maintaining physical activity for cancer survivors, particularly the support of other survivors [39]. Feelings about their capability also reflected the importance of the social environment. A key barrier to activity was the comparison to healthy individuals as well as concern about what others might think about their abilities and body. Therefore, tailoring physical activity programmes for women with MBC only could provide a more comfortable environment, and offer important social support to help engage in activity.

### 4.2 Study Limitations

Our study had limitations. We asked participants to self‐report whether they had MBC but did not verify with hospital records. We may have incorrectly included non‐MBC participants. The diversity of our sample could have been improved to explore demographic differences in physical activity beliefs, barriers and facilitators. Despite attempting to recruit individuals from diverse backgrounds, participants were mainly White British and University educated, with research in the metastatic population suggesting these groups are more likely to be active [16]. Similarly, our sample may have been partly self‐selecting. Those more interested in physical activity may have been more likely to take part, which could explain the mostly positive attitudes towards physical activity. This may have limited the generalisability of our study to diverse groups. Further research should specifically explore the needs and challenges of underserved and less active groups to ensure any intervention is sensitive to the needs of a diverse population. As is common in qualitative studies, the positionality of the researchers, in this case our positive attitudes to physical activity, is likely to have affected the results. Despite strengthening the link between our findings, theory and future intervention design, mapping our findings onto the TDF may have constrained our implications. Finally, we conducted telephone interviews only, which may have affected the interviewer‐participant dynamic. Our first interviews were conducted when some COVID‐19 measures remained in place in UK universities and as our participants were often immunocompromised, we did not meet in‐person. Telephone interviews were chosen over video conferencing to help reach a diverse group of participants, some of whom may have lacked internet access or confidence.

## 5 Conclusion

Our UK study highlighted that women with MBC want to be active but experience a range of physical, psychological and healthcare barriers. We highlighted a lack of appropriate support from healthcare providers, with participants expressing a need for more tailored, sensitive and holistic approaches which consider their unique needs. Our study was the first in this area to utilise theory, and in doing so, we highlighted several implications for future interventions to support physical activity in women with MBC. We encourage clinicians to implement physical activity recommendations with their patients, to improve knowledge about the benefits and risks of physical activity and in the future, incorporate psychological and social support into physical activity interventions. We highlighted again that the MBC population is diverse, with different clinical and demographic profiles, physical capabilities, and preferences. Offering tailored and holistic physical activity support which considers these unique needs is likely to be effective at improving activity levels in this population. Given the potential benefits of physical activity for the quality of life of women with MBC, future work should consider cost‐effective ways of delivering this, ensuring the needs of all patients are met.

## Author Contributions

Z.F.H. led study conceptualisation, methodology, project administration, recruitment and data collection, supported by R.J.B., S.G.S. formal analysis was led by Z.F.H. and supported by A.A.‐S., K.E.L. supported the development of study materials and analysis plan. The initial draft of the manuscript was written by Z.F.H., S.G.S., R.J.B., A.A.‐S., and K.E.L. reviewed and edited draft versions and approved the final manuscript. The project was supervised by S.G.S. and R.J.B.

## Ethics Statement

This study was performed in line with the Declaration of Helsinki and was approved by School of Medicine Research Ethics Committee at the University of Leeds (MREC 21–040). Ethical approval for the ASCOT study was obtained from the National Research Ethics Service Committee South Central—Oxford B (14/SC/1369).

## Conflicts of Interest

Smith has received consulting fees from Lily. The remaining authors have no relevant financial or non‐financial interests to disclose. The views expressed in this publication are those of the author(s) and not necessarily those of the NHS, the National Institute for Health and Social Care Research or the Department of Health and Social Care. The funders had no role in the study design, data collection, analysis, interpretation of data, and in the writing of this manuscript.

## Supporting information

Supporting Information S1

Supporting Information S2

## Data Availability

The interview schedule is included in the Supplementary Materials. Restricted access to a subset of pseudo‐anonymised interview transcripts is available from the University of Leeds Research Data Repository (https://doi.org/10.5518/1680). Only the transcripts for participants who consented have been uploaded to the repository.
