# Supplementary material for: ‘As Important as Medication’. A Qualitative Investigation of the Beliefs, Barriers and Facilitators of Physical Activity for Women With Metastatic Breast Cancer
Source: Psychooncology. 2025 May 28;34(6):e70193. doi: 10.1002/pon.70193 (PMC12120048; doi:10.1002/pon.70193)
Supplement: Supplementary file 1 — Supporting Information S1 [file PON-34-e70193-s001.pdf]

Telephone Interview Study

Physical Activity in Women with Metastatic Breast Cancer

Interview Schedule

Welcome, introduction, aim of the study, process of participating

Thank you for agreeing to take part today. Before we start the interview, I want to quickly go over some key points about your rights as a participant. Some of the topics that we will be discussing might be sensitive in nature, but please know that I will not judge you in any way. It's completely fine if at any point you would like to stop the interview, or if you do not want to answer a question, please just let me know. You can also withdraw from the study at any point, and you do not have to provide a reason for why. You can also withdraw any identifiable data at any point but your non-identifiable information will be kept as the research is being conducted in the public interest.
Just to remind you the interview will be recorded, but anything you say to me today will be kept confidential, and if you are quoted in any of our reports we'll use a fake name, and anonymise anything identifiable you mention as well.

After the interview, if you have any further questions about anything we discussed today, please feel free to contact me. However, if you have any questions about cancer or your specific medical case, please refer directly to your doctor as I am not able to answer medical queries.

Permission to record

- Ask permission to record the conversation.
- Turn the recorder ON to confirm their name and to obtain verbal consent. Then turn the recorder OFF and ON again to record the interview section anonymously.

Consent Statement

I confirm that I have received, read and understood the Participant Information Sheet and the Privacy Notice. I confirm that I am willing to participate in this telephone interview where I will be asked questions about my cancer and about my physical activity. I understand that my participation in this study is voluntary. I can withdraw from the study at any time without giving reason or justification and without my medical care or legal rights being affected. I therefore confirm that I am happy with the information that has been provided and consent to this interview.

If consent is provided, start the interview

If you agree, please could you say your name and the date?

I will now turn the recorder off and on again so that the rest of the transcribed interview and your responses remain anonymous.

Outline of the study and definition of key terms

This interview should take around 45 minutes, but it could be shorter or longer, depending on what you want to talk about!

The aim is to discuss your thoughts about and experiences with physical activity as someone with advanced cancer, as well as what you might like to have, or not have, in a future programme. Just to confirm, by physical activity we mean anything that involves you moving about. So, this can be like low-intensity things like walking, or even gardening and household tasks, to more intensive exercise like running or swimming, in other words, things that get your heart beating.

1) **Physical activity levels before/after cancer diagnosis**
   - Firstly, can you tell me a bit about your physical activity levels before you were diagnosed with cancer?
     - **Prompt**: What kind of things did you do to try and keep fit?
   - What about when you were first diagnosed with cancer?
     - What about during treatments?
     - What about now?

2) **Physical activity Beliefs**
   - How do you feel about your physical activity levels now?
   - How do you feel about trying to be more active?
     - **Prompt:** Is it something you want to do or not want to do?
   - If yes, what ways have you tried to increase your activity levels?
     - **Prompt:** What method/route do or did you prefer?
     - Why did you prefer this?
   - If you are trying to be active, what effects, if any, does physical activity and exercise have on your cancer symptoms?
     - general physical health?
     - wellbeing?

3) Barriers and facilitators to physical activity

- What do you think are main challenges to increasing and maintaining your physical activity levels?
  - **Prompt:** physical challenges, logistical challenges, motivation/wellbeing

4) Physical activity support

- Has a healthcare professional or someone else recommended you try and increase your physical activity, as someone with advanced cancer?
  - If yes, what did they recommend?
  - Did you take their recommendations?
- What kind of support would you like to have to increase and maintain your physical activity levels?

5) Preferences for physical activity

I'm now going to present some scenarios to get your thoughts about different types of physical activity programmes

- What are your thoughts on the following physical activity scenarios?
  - home-based vs gym-based exercise
  - group vs lone exercise
  - supervised vs unsupervised exercise
- Say that some researchers were looking to develop a programme to increase physical activity levels specifically for patients with metastatic cancer, what would you like to see in that intervention?
  - What would you like to NOT see in that intervention?

Next, I'm going to ask you some questions about digital health applications that some people use to try and increase their physical activity.

- To start, have you come across or used digital devices to try and increase your physical activity? For example, mobile apps, fitness watches or trackers.
  - *If already mentioned, pick back up on that*
- If yes, what was your experience with using them?
- If no, would you consider using them in the future?

- why?

Aims of physical activity research

Finally, I wanted to get your thoughts on wider research for patients like you, with advanced cancer. This work is part of a broader programme of research that would like to test a physical activity programme in people with advanced cancer. But to do this, we need to be really clear about what we want to test and why. So, for your personally, what do you think the main goal of physical activity research with patients like you should be?

- **Prompts**
  - to help you live better
  - to help you live longer
  - to help you manage stress/improve wellbeing
  - to help manage symptoms
  - to help improve strength
- what do you think researchers should be looking at?

Debrief

Thank you so much for your answers today. Before we end:

- Is there anything else I haven't asked about that you think is important?
- Is there anything else you'd like to ask me?

If you would like to ask me further questions on the study later on, please do not hesitate to contact me. I'm going to email you a debrief form as well as a brief demographic questionnaire. I would really appreciate if you could send this back to me when you get chance.

Thank them for their time.
