# Supplementary material for: ‘As Important as Medication’. A Qualitative Investigation of the Beliefs, Barriers and Facilitators of Physical Activity for Women With Metastatic Breast Cancer
Source: Psychooncology. 2025 May 28;34(6):e70193. doi: 10.1002/pon.70193 (PMC12120048; doi:10.1002/pon.70193)
Supplement: Supplementary file 2 — Supporting Information S2 [file PON-34-e70193-s002.pdf]

3 Results

Our analysis generated five main themes: (1) Positive beliefs and knowledge about physical activity; (2) Physical ability limits physical activity; (3) Important psychological aspects of physical activity; (4) Levels of support from HCPs and charities and (5) Making physical activity more accessible. Table 2 provides an overview of these themes and their sub-themes, mapped onto TDF domains.

Table 2: Overview of themes and sub-themes, mapped onto the Theoretical Domains Framework (TDF)

Main Theme	***Sub-themes***	***Main TDF Domain(s)***
**3.1 Positive Beliefs and Knowledge about Physical Activity**	Recognising the importance of physical activity	Intentions
	Experiencing the benefits of physical activity	Beliefs about Consequences<br>Reinforcement
	Having knowledge about physical activity	Knowledge<br>Beliefs about Consequences
	Having a balanced view of physical activity in life	Optimism
**3.2 Physical Ability Limits Physical Activity**	Physical symptoms limit physical activity	Environmental Context and Resources<br>Skills
	Diversity of experiences affected physical activity levels	Environmental Context and Resources
	Fearing an injury limits physical activity	Emotion<br>Beliefs about Consequences<br>Beliefs about Capabilities
	Fearing an infection limits physical activity	Emotion<br>Beliefs about Consequences
**3.3 Important Psychological Aspects of Physical Activity**	Poor mental health as a barrier	Emotion
	Lack of confidence about capabilities and body image limits physical activity	Beliefs about Capabilities<br>Emotions<br>Social Influence
	Having low motivation to exercise	Intentions<br>Goals<br>Optimism
	Physical activity helps with wellbeing	Emotion<br>Beliefs about Consequences<br>Reinforcement
	Importance of psychological support for physical activity	Social Influence<br>Environmental Context and Resources
	Importance of social support for physical activity	Social Influence
**3.4 Levels of Support from HCPs and Charities**	Lack of physical activity support from HCPs	Environmental Context and Resources<br>Knowledge
	Supportive HCPs facilitates physical activity	Environmental Context and Resources
	Charities play a key role	Environmental Context and Resources

**3.5 Making Physical Activity more Accessible**	Bringing physical activity into the day makes it easier to maintain	Intentions<br>Behavioural Regulation
	Making physical activity fun helps with motivation	Social Influence
	Setting reasonable goals helps maintain physical activity	Goals
	Need for tailored support which is specific for MBC	Environmental Context and Resources
	Need for tailored support which accounts for individual differences	Environmental Context and Resources
	Physical activity should be considered within a holistic approach to health	Environmental Context and Resources

3.1 Positive beliefs and knowledge about physical activity

Most participants believed physical activity was important and they tried to remain active.

"Physical activity needs to become as important as whatever medication you give to a cancer patient." – RT

"So I think definitely exercise, definitely helped me during the darker times. It would be very important to me; I would feel devastated if it got to the stage where I couldn't do it." – OR

One key reason was experiencing the benefits of being active. Participants reflected on how physical activity helped with their symptoms and treatment side-effects, for example, with energy, strength, sleep, digestion, and muscle pain.

"When I was doing my jogging group, I used to be on a real high afterwards…I'd have so much energy, even though I'd just ran." - KF

Participants also discussed their knowledge and beliefs about physical activity in the context of cancer, for example preventing recurrence and promoting longevity.

"I stick to this training programme quite a lot…probably because I think that this keeps my cancer at bay." – ZL

"I was under the impression that keeping physically active would help prevent its reoccurrence" – FG

However, many reflected on the balance of physical activity in their lives. Participants noted it was not the most important thing, nor should they feel guilty for struggling.

"Our life is so up and down and it's so unpredictable that it's hard to commit to something when there are already lots of commitments…it's not the most important commitment. (They should) not feel guilty if they can't do it or are too tired or have got something more important on." - EB

3.2 Physical ability limits physical ability

Participants were diverse in their physical capability levels. Some reported no limits, but most discussed struggling to some degree with being active. Participants consistently reported physical symptoms made activity more challenging. Fatigue and pain were most commonly reported, but breathlessness, neuropathy, muscle stiffness, lymphoedema, hand and foot syndrome and nausea were all discussed.

"I would get a lot of joint pain so sometimes I would struggle to walk, even getting out of bed would be hard…I did suffer from very sore knees, very sore ankles and sore elbows and so just doing physical things was quite painful at times." – EB

"Sometimes I don't even want to go out because it's so difficult. I have to make sure I am going somewhere I can stop and sit down because as I walk I need to sit and breathe. So I have to choose where I am going because if it's a long walk I'm going to have problems." – GR

"I think you just lounge about because you feel, because of the chemo you feel sick all the time, you feel vomity, nauseated, so you're mostly concentrating on that, or you try to put yourself to sleep that you don't feel those symptoms." – LO

There was diversity in participant treatment experiences depending on what they received, or disease progression. Physical symptoms appeared worse during chemotherapy. Moving onto treatments with fewer side effects was a facilitator of physical activity. Those living with MBC for longer also noticed a gradual decrease in their physical abilities.

"(My cancer) has progressed steadily… I am constantly on treatment. So, my energy levels and my pain levels by now have markedly deteriorated and stop me being active." - RW

Physical symptoms contributed to health-related fears. Many were afraid they would injure themselves because they were recovering from surgery or had bone metastases.

"I just withdrew into myself…I didn't feel that I could (exercise) because I was scared of hurting myself…Now I'd like to pick up more activity, but I'm scared." – RG

Additionally, because their cancer treatments compromised their immunity, some felt anxious about attending indoor exercise classes, going to gyms or swimming, fearing an infection.

"But I haven't gone back to that because I have been worried about Covid really which has put me off…I don't really want to get too close to people so I would say Covid has really affected what I haven't gone back to." - FG

3.3 Important psychological aspects of physical activity

Many participants discussed the devastating effects of their diagnosis on their mental health, which acted as a barrier.

"There's a limit to what I could have done even if I'd been physically well enough to do it...Mentally you're down because of the whole shocking situation…to be told it was very limiting at the time was pretty tough to deal with, so physical activity wasn't in the remit." - TE

Their reduced physical ability made them feel upset, disheartened, embarrassed and angry. Many found themselves comparing their ability to pre-diagnosis or to other healthy women, reducing their desire to exercise in public. They talked about a lack of confidence and body image, especially if they had undergone a mastectomy. Not seeing representation of women who looked like them made them feel gyms and exercise classes were not for them.

"It was a bit disheartening. I ended up stopping that yoga class, because I felt mentally it was making me feel bad, that I couldn't do the things that I did before." - DG

"I would feel obligated to wear my prosthetics and it's uncomfortable to exercise wearing false boobs…When you're in a gym, you're wearing tight-fitting clothing. There's no hiding." - RW

As a result, low motivation to engage in activity was highlighted. Physical activity also felt harder, so some did not enjoy high-intensity exercise. Not being able to meet their goals acted as a barrier.

"I'm struggling with trying to motivate myself to do things because everything seems so much of an effort really when you're suffering with fatigue and feeling quite down." - GH

However, nearly all women agreed being active supported their wellbeing, for example helping with stress, difficult feelings about their diagnosis and to feel 'normal'. The combination of physical activity and being outdoors in nature was highlighted by a significant proportion of participants. The strong, positive effects physical activity had on wellbeing acted as a facilitator, as they knew it would make them feel better.

"I would feel proud of myself after and it would lift my spirits a little bit…That's why every day I'm so keen to just try and do something, because I would feel like I would be losing everything if I lost that as well." – EW

"If I go out for a walk, and we go somewhere where there's, you know, grass and greenery, it- and you're hearing the birds singing, it makes me just think about where I am, and I'm looking for the birds, or I'm listening to the birds, and it takes my mind completely away from my illness." - RT

Consequently, psychological support also acted as a facilitator with supportive environments helping them to overcome psychological barriers.

"Because actually, your mind is probably a bit more powerful than your body because it influences, doesn't it? So I don't know if that would come within the remit, but I just think psychological input is quite important, probably more important than we give it credit for." - RG

Social support was consistently highlighted as a way to make physical activity feel less like a 'chore' and address isolation, especially with others who understood what they were experiencing.

"Social support and physical activity combined, I think could be a really good call… so people feel supported together, but at the same time, seeing the physical activity worthwhile." – CS

"The community aspect of exercise is also helpful mentally and it, because I know it's just, well our classes were a good laugh, they were fun, whereas sometimes doing things at home seems like a chore." - GH

3.4 Levels of Support from HCPs and Charities

Nearly all participants noted they did not receive enough support with physical activity, and their healthcare provider (HCP) did not recommend it. Some reported their HCP actively discouraged physical activity because of risk of injury. Local and accessible opportunities to engage in physical activity appeared difficult to find which made them unsure about what they could do.

"I was scared of doing too much physical activity because I just didn't get any good advice from any of my care team…when I asked what I could do, physical activity-wise, it was just, "Oh, don't know, probably best not to do anything". – PD

"(I asked) whether I can carry on with what I'm doing and when I was on chemo the answer was, well no not really, and that's about as far as it's gone in terms of discussion. There was no sort of alternative or any other suggestions made." - GH

Participants believed physical activity should be encouraged by HCPs, and support provided by NHS. Wider discussions were held about the cancer care system, with some feeling like they were only treated as a physical diagnosis.

"There's nothing. You know, you're just left alone. There's no sort of like…bedside manners? So there should be more programmes, more discussion so people know what they're going through." - LO

Despite the majority feeling they had not received enough support, some participants reflected on good examples of care, which facilitated physical activity.

"The nurse that I saw just said, "It is really important that you do some physical activity, it doesn't matter what it is…so that your mind is not just going over and over what is wrong with you," and I found that very helpful advice." - RT

Additionally, the role of charities were positively highlighted in encouraging physical activity which appeared to fill a much-needed hole in service provision.

"Penny Brohn (charity) feel strongly about it when the hospitals don't. They medicate you and they keep, monitor you, but they don't offer you or suggest things which would make your life easier and better. You rely on charities to help you with that sort of thing." – GH

3.5 Making PA more Accessible

Participants discussed ways that physical activity could be made easier and accessible. First, by making it a part of their day, such as going on the school run, gardening, housework, walking to the hospital, or walking the dog. These activities were easier to maintain as they became routine.

"(It's) suggesting other ways of getting physical exercise. I've joined a local gardening group that does community gardening and makes our local area look nice, so I'm actually getting physical exercise through doing." - EB

Participants who struggled with motivating themselves suggested making physical activity fun, for example by combining it with socialising, music or a visit to a coffee shop.

"So it's, yes, but fun, make it fun and so you don't feel like you are doing exercise, so yes, just make it enjoyable really." - FG

Some reflected on setting reasonable goals and tangible plans. Making plans to be active, helped participants to remain active. Several participants owned a smart watch or a phone app which was used to set goals. Setting their own goals which considered their complex needs was helpful, but when goals were unattainable, they had the opposite effect.

"A small goal that you can perhaps reach and then move on to the next goal, it certainly does help you." – EW

"My friend bought me a Fitbit thing a few years ago but I didn't like it because it just kept telling me that I wasn't doing enough steps so I found it quite demotivational really, and it makes it more into a chore." - FG

Nearly all participants discussed the need for tailored support, reflecting on the unique and diverse experiences of women with MBC. This was two-fold. First, patients reported a lack of specific service provision for women with MBC specifically. General services were not sufficient, and even cancer-specific programmes often ignored the unique needs of secondary cancer.

"Most personal trainers, Pilates teachers, yoga teachers do not really understand the limitations of people with any kind of metastatic cancer because that often goes to the bones…(they) do not seem to understand the consequences of that." – RW

"But then nobody really talks about secondary, or stage four and it comes as a terrible shock. I understand that they don't want to tell people with primary, this could happen because it's scary stuff. But on the other hand, there doesn't always seem to be a lot of awareness. When I went to that thing at the hospital that was for primary and secondary patients, it was really, nobody really knew what secondary breast cancer was" – EW

Second, participants expressed need for support which accounted for the differences within the MBC population, reflecting on the diversity of capabilities, and how needs change over time. Staged programmes which had different options depending on the ability of the individual were mentioned by several participants.

"(Charities) have got somebody trained to tutor people with different levels of ability due to cancer and they're very good at offering alternative types…They usually demonstrate something at a lower level for those who haven't got the same amount of movement capabilities." – GH

"I think it definitely needs to be something, you know, staged where we have you know, things that can be accessible, I suppose like the yoga scenario, you know, do as little or as much as you can." - DG

Finally, conversations about tailored interventions brought up wider discussions about taking a holistic approach to their health. Participants argued that physical activity should be offered as a core part of treatment, along with other lifestyle and psychological support.

"(We need to) treat people in holistic ways rather than just a lump of meat with a pain…Yes, we have to have medications and drugs but incorporate a little sort of plan for the individual, because that's what we need." – OR
